# Differentiating the Structural and Functional Instability of the Craniocervical Junction

**DOI:** 10.3390/healthcare12192003

**Published:** 2024-10-07

**Authors:** Piotr Godek, Wojciech Ruciński

**Affiliations:** Sutherland Medical Center, 04-036 Warsaw, Poland; rucinskiwojtek@gmail.com

**Keywords:** chronic neck pain, chronic instability, craniocervical junction, diagnosis, symptomatology, clinical outcomes, quality of life

## Abstract

This paper presents the anatomical and biomechanical aspects of chronic instability of the craniocervical junction (CCJ) with a discussion on clinical diagnostics based on mobility tests and provocative tests related to ligamentous system injuries, as well as radiological criteria for CCJ instability. In addition to the structural instability of the CCJ, the hypothesis of its functional form resulting from cervical proprioceptive system (CPS) damage is discussed. Clinical and neurophysiological studies have shown that functional disorders or organic changes in the CPS cause symptoms similar to those of vestibular system diseases: dizziness, nystagmus, and balance disorders. The underlying cause of the functional form of CCJ instability may be the increased activity of mechanoreceptors, leading to “informational noise” which causes vestibular system disorientation. Due to the disharmony of mutual stimulation and the inhibition of impulses between the centers controlling eye movements, the cerebellum, spinal motoneurons, and the vestibular system, inadequate vestibulospinal and vestibulo-ocular reactions occur, manifesting as postural instability, dizziness, and nystagmus. The hyperactivity of craniocervical mechanoreceptors also leads to disturbances in the reflex regulation of postural muscle tone, manifesting as “general instability”. Understanding this form of CCJ instability as a distinct clinical entity is important both diagnostically and therapeutically as it requires different management strategies compared to true instability. Chronic CCJ instability significantly impacts the quality of life (QOL) of affected patients, contributing to chronic pain, psychological distress, and functional impairments. Addressing both structural and functional instability is essential for improving patient outcomes and enhancing their overall QOL.

## 1. Introduction

The craniocervical junction (CCJ) involves more than just the connection between the skull and the first cervical vertebra via the C0/C1 joint. It broadly encompasses the upper cervical segment from C0 to C2/C3, which is crucial for several reasons:

(1) The anatomical aspect: A significant part of ligamentous stabilization for the C0/C1 level originates at the C2 segment (alar and accessory alar ligaments) with the coordinated movement of the C0/C1/C2/C3 segments in lateral flexion and rotation.
(2) The neurophysiological aspect: The range of the spinal nucleus of the trigeminal nerve and the innervation of the suboccipital muscles reaching the C3 segment result in numerous clinical implications (certain types of headaches and dizziness and some temporomandibular joint dysfunctions).
(3) The vascular aspect: The course of the vertebral arteries in their V3 and V4 segments is between the foramina in the transverse processes and enters into the foramen magnum (two critical bends between C2/C3 and C1/C2) [1,2,3].

The complex multi-layered ligamentous capsular system is the main stabilizer of the CCJ, as at no level does the articular surface configuration alone provide stabilization, and the paraspinal muscles closest to the axis of rotation form tiny structures that are more involved in a proprioceptive function than a protective one [4,5].

The primary stabilizing role of the C0/C1 level is played by the anterior and posterior atlanto-occipital membranes, joint capsules, and the tectorial membrane, with the alar, accessory alar, and apical ligaments from the C2 segment providing important supplementary support. For the C1/C2 level, the primary stabilizer is the transverse ligament, part of the cruciate ligament, which complements the ring around the odontoid process, with additional bands of the alar ligaments from the C2 base further reinforcing the entire C0/C2 complex [6,7,8].

Loss or insufficiency (laxity) of these elements can lead to CCJ instability. However, CCJ instability eludes the classic definition of instability proposed by Panjabi and White. According to these authors, this phenomenon involves the spine’s inability to maintain proper relationships between vertebrae under physiological load forces without causing damage to neural structures such as the brainstem, spinal cord, or nerve roots [9].

In the case of CCJ instability, neurological symptoms associated with the compression of the brainstem, medulla oblongata, and spinal and cranial nerves are possible. When combined with vertebral artery compression, they result in very rich and often difficult-to-differentiate symptomatology. The morphological basis of CCJ instability involves the following: (1) osseous articular defects, including congenital anomalies (Chiari malformation), traumatic injuries to articular surfaces, rheumatoid arthritis (RA), infections (tuberculosis), and Paget’s disease; (2) ligamentous defects, including congenital ligamentous laxity (Ehlers–Danlos syndrome, Down syndrome, Loeys–Dietz syndrome, Stickler syndrome, Marfan syndrome, cleidocranial dysostosis, and Morquio syndrome), traumatic ligamentous injuries (direct or indirect mechanism), and RA; (3) myofascial defects, including muscle balance disorders, postural syndromes, dystonias; and (4) neurogenic defects, including dysfunction of the cervical proprioceptive system (CPS) with impaired coordination of stimulation from cerebellar, vestibular, and visual centers (“proprioceptive storm”) similar to those following whiplash injuries or dystonias [10,11,12].

Therefore, CCJ instability can be divided into true (structural) and false (functional) cases. In the first case, significant elongation or damage to the ligamentous complex or the deformation of osseous structures occurs, leading to abnormal movement patterns in mobility and provocative tests as well as changes in radiological images. In the second case, no significant abnormalities are found in clinical or radiological tests, but there is predominant muscle hypertonia, kinesiophobia, pain and dizziness, balance disorders, pseudo-radicular and pseudo-meningeal symptoms, dysautonomia, dysphoria, mood and concentration disorders, and many other symptoms indicating impulse dysregulation in the nervous system; this is comparable to “informational noise” rather than true instability.

This paper aims to draw the medical community’s attention (including physicians, osteopaths, and physiotherapists) to the existence of an atypical form of instability in the upper cervical spine (CCJ). This form of instability, referred to by the authors as functional or false instability, is characterized by the absence of structural damage (such as ruptures, elongations, tears, or insufficiency) in the highly complex ligamentous capsular system of the CCJ. Moreover, it does not manifest in imaging studies, which typically provide measurement criteria for structural damage within the CCJ’s ligamentous capsular system. Functional instability is the result of disrupted perception, processing, and coordination of signals from peripheral proprioceptive receptors (which are abundantly represented in this region) by the central nervous system. This leads to the sensitization of peripheral receptors located in the paravertebral muscles, joint capsules, ligaments, connective tissue membranes, and dura mater, as well as the spinal cord (including cranial nerve nuclei, e.g., the spinal nucleus of the trigeminal nerve) and other centers in the central nervous system. This occurs under the influence of stimulus facilitation, with a simultaneous deficit in feedback inhibition. As a result, the complex system of information exchange between the proprioceptive, vestibular, cerebellar, oculomotor, and vasomotor systems becomes destabilized [13,14]. Clinically, this may present with symptoms similar to those seen in true (structural) instabilities of the CCJ, which are often associated with the compression of specific nerve or vascular structures and may require surgical intervention. However, unlike true structural instability—where the protection of sensitive neural and vascular structures from compression and ischemia through decompression and surgical stabilization is necessary—functional instability can and should be treated with conservative methods.

## 2. Diagnostics and Etiology of CCJ

The diagnosis of CCJ instability begins with a medical history and clinical examination using tests derived from manual therapy. These tests are divided into two categories. The first category includes mobility and tissue compliance tests, which are used to assess mobility dysfunctions in the osteopathic sense. Identifying such dysfunctions determines the method of manual soft tissue treatment to be used with the aim of restoring proper joint play and postural re-education of head malposition, which usually accompanies functional forms of CCJ instability. The second category consists of provocative tests, which are employed during the clinical examination to detect important and potentially dangerous ligamentous deficits that could ultimately lead to compression or secondary damage to critical neural or vascular structures. Even one positive provocative test is a contraindication for manual therapy and serves as an indication for a medical consultation with osteopaths or physiotherapists. Confirmation of structural damage in the form of displacement of reference points in radiological measurements serves as an additional criterion supporting the presence of true CCJ instability. Bearing in mind the above-mentioned criteria, we can recognize three types of CCJ instability:

- Ligamentous-Capsular Hypermobility: This condition is characterized by an increased range of motion due to excessive laxity in ligamentous capsular structures without associated damage. It typically presents with non-specific neck pain, occasional discomfort, or mild instability but without significant neurological symptoms or imaging findings indicative of structural damage [15].
- Functional Postural Disorders (Proprioceptive Dysfunction of the CCJ): Functional instability arises from a disruption in the proprioceptive system’s ability to accurately perceive and control cervical spine movement. Despite the absence of structural damage on imaging, patients may experience symptoms such as dizziness, balance issues, and muscle hyperactivity. This form of instability requires careful differentiation from structural causes as it can mimic neurological and musculoskeletal dysfunctions. Treatment typically involves conservative measures such as proprioceptive retraining and physical therapy [16].
- True Structural Instability: This condition involves an actual disruption of ligamentous capsular structures, such as tears, elongations, or ligament insufficiency. It is usually confirmed through imaging techniques and often requires surgical intervention to prevent further neurological damage or instability. Patients typically present with clear signs of instability, such as neurological deficits, significant pain, and radiographic evidence of vertebral misalignment or ligament damage [17].

## 3. The Biomechanics of the CCJ and Manual Examination

To distinguish true from false CCJ instability, it is essential to understand the biomechanics of this segment and the resulting clinical and provocative tests.

**Figure 1:**
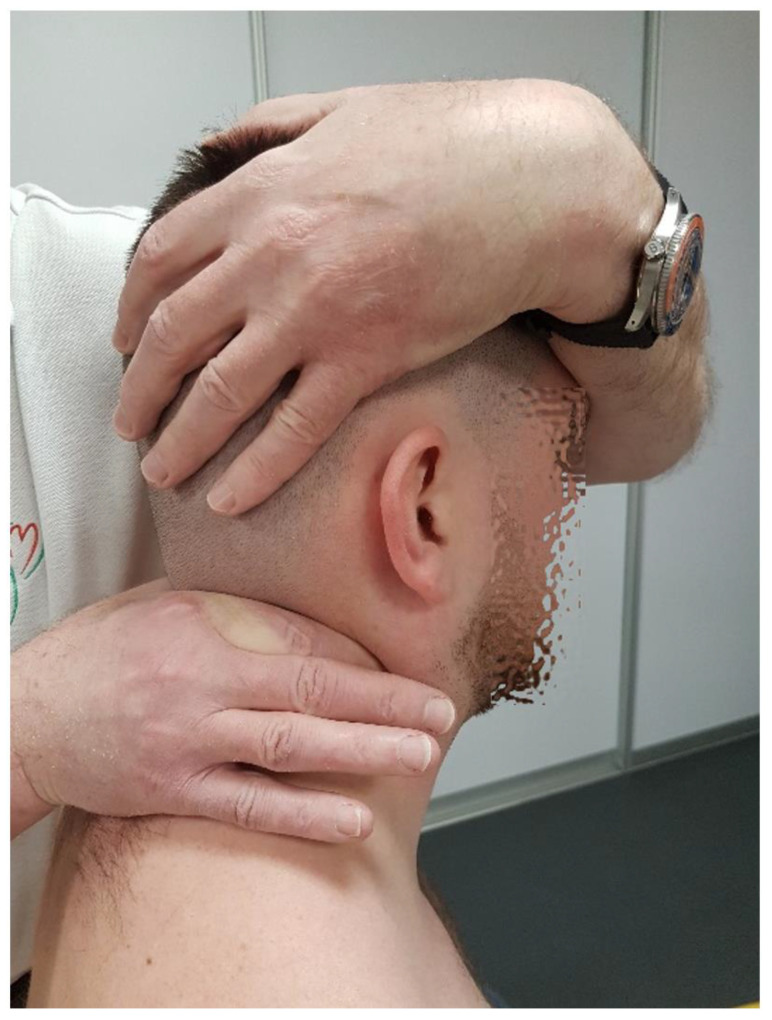
Flexion–extension mobility test for C0/C1 level.

**Figure 2:**
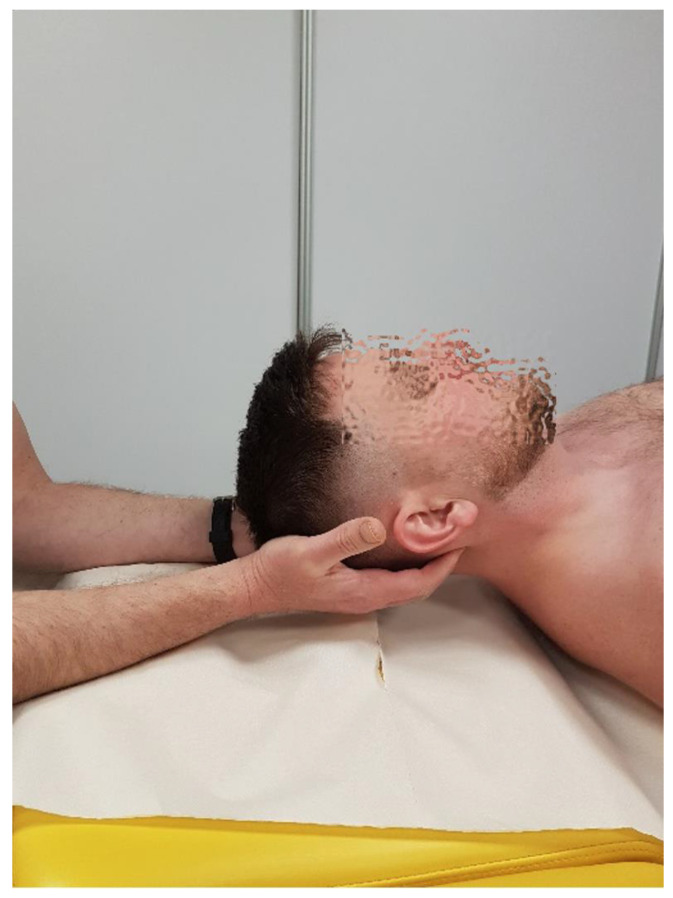
Rotational mobility test for C0/C1 level.

**Figure 3:**
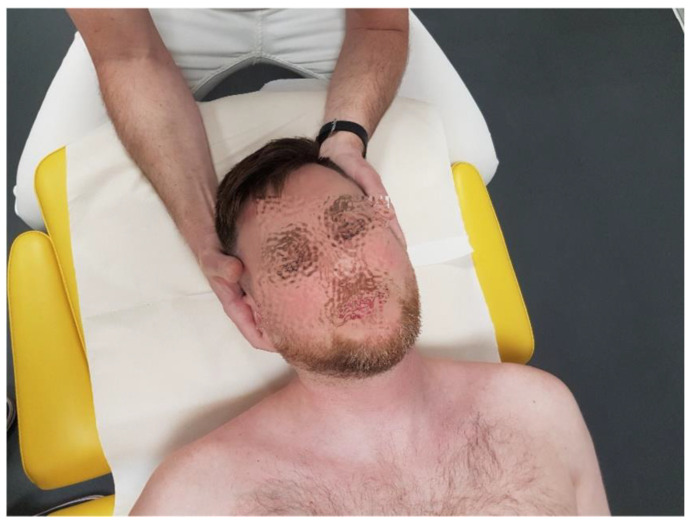
Lateral mobility test for C0/C1 level.

At the C0/C1 level (atlantooccipital joint, AOJ), the following movement components are possible:

- Flexion/Extension: This refers to the anterior and posterior glide of the occipital condyles on C1, totaling about 30° (10° in flexion and 20° in extension, constituting about 17% of the cervical spine’s flexion range); movement occurs around an axis passing through the external auditory meatuses [18]. The manual testing of the glide range at this level is performed with the patient seated. The therapist first stabilizes the atlas arch by placing one hand over the posterior aspect of the C1 transverse processes. With the other hand, the therapist stabilizes the patient’s head by placing their palm over the occiput and using their fingers to control the mandible. The head is then passively moved in the sagittal plane, maintaining alignment along an axis passing through the external auditory meatuses, with careful application of flexion and extension. The therapist assesses the range of motion (normal range: 10° flexion, 20° extension), tissue compliance, and end-feel resistance using repetitive movements to ensure consistency in the findings (Figure 1).

- Rotation: This refers to movement by approximately 5–7° to each side; the movement is coupled with lateral flexion due to the configuration of the C1 articular surface and the forces exerted by the alar ligaments. The manual testing of the glide range at this level is performed with the patient in a supine position. The therapist elevates the atlas arch through its transverse process and assesses the range, quality of motion, and end-feel resistance of the relative posterior glide of the occipital condyle on the same side (Figure 2).

- Lateral Flexion: This refers to movement by approximately 3–5° to each side; the movement is coupled with a component of contralateral rotation at the C1/C2 level due to the action of the alar ligaments, making it difficult to isolate (coupled movement extends to C2/C3). For example, lateral flexion to the right causes C1 to rotate in the opposite direction and manifests as increasing tension on the C1 transverse process (it appears to protrude and “harden” during the lateral glide of C0, with the alar ligament on the opposite side to lateral flexion providing resistance) [19] (Figure 3).

A confirming test for true instability at the C0/C1 level is the blurring of the end-feel resistance during mobility testing in the three described directions or an increase in the range of lateral flexion without the physiological component of rotation (deficit in alar ligament function), as well as a positive lateral shear test with a blurred (soft) end-feel.

At the C1/C2 level (atlantoaxial joint, AAJ), only a rotational movement component is possible:

- Flexion and lateral flexion: 0°.
- Rotation: 30–40° to each side (comprising approximately 50% of the cervical spine’s rotational range). Isolation of rotational movement at this level typically occurs after full flexion or extension of the lower cervical spine. Coupled contralateral lateral flexion is also observed—for example, during right rotation, the C1 transverse process on the same side elevates [20].

**Figure 4:**
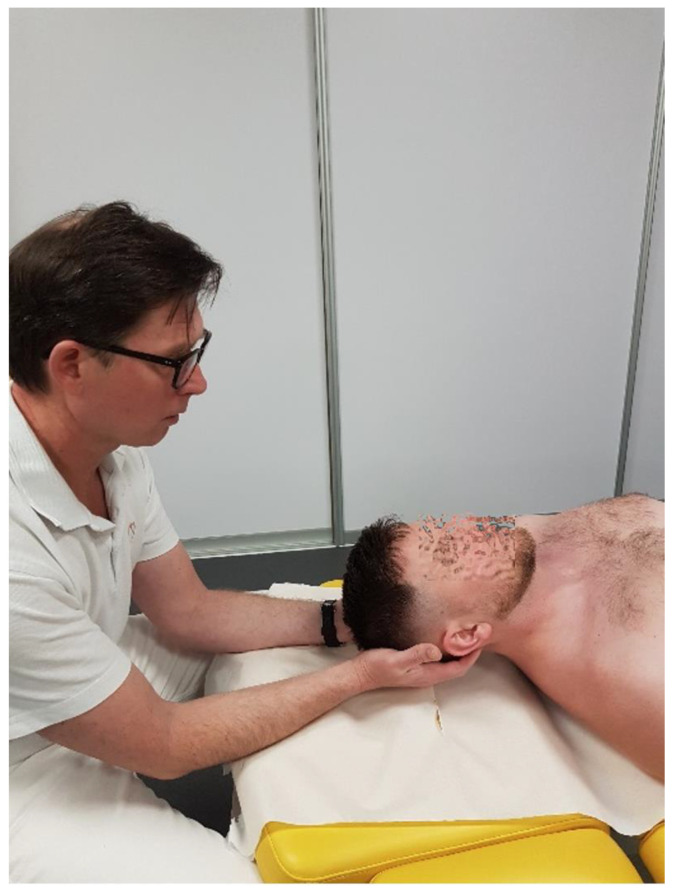
Passive provocative test for anterior instability at C1/C2 level (Anterior Shear Test).

To detect true C1/C2 level instability due to transverse ligament damage, two key tests are commonly employed:

The Anterior Shear Test: This test is performed with the patient in a supine position. The therapist begins by stabilizing the C2 spinous process with one hand to prevent its movement. With the other hand, the therapist gently grasps the transverse processes of C1 and applies a slow, controlled anterior glide. This motion is carried out to assess for any abnormal anterior movement of C1 relative to C2 while monitoring end-feel resistance. Excessive anterior displacement or a soft end-feel may indicate transverse ligament insufficiency or instability (see Figure 4 for a visual representation of the test) [21].

The Sharp Purser Test: This test is performed with the patient seated. The therapist asks the patient to flex the head forward slightly while applying resistance to the forehead with one hand. Simultaneously, the therapist stabilizes the C2 spinous process with the other hand. A characteristic “clunk” sound or sensation of C1 moving posteriorly over C2 during the test suggests C1/C2 subluxation, indicative of transverse ligament damage (Figure 4).

**Figure 5:**
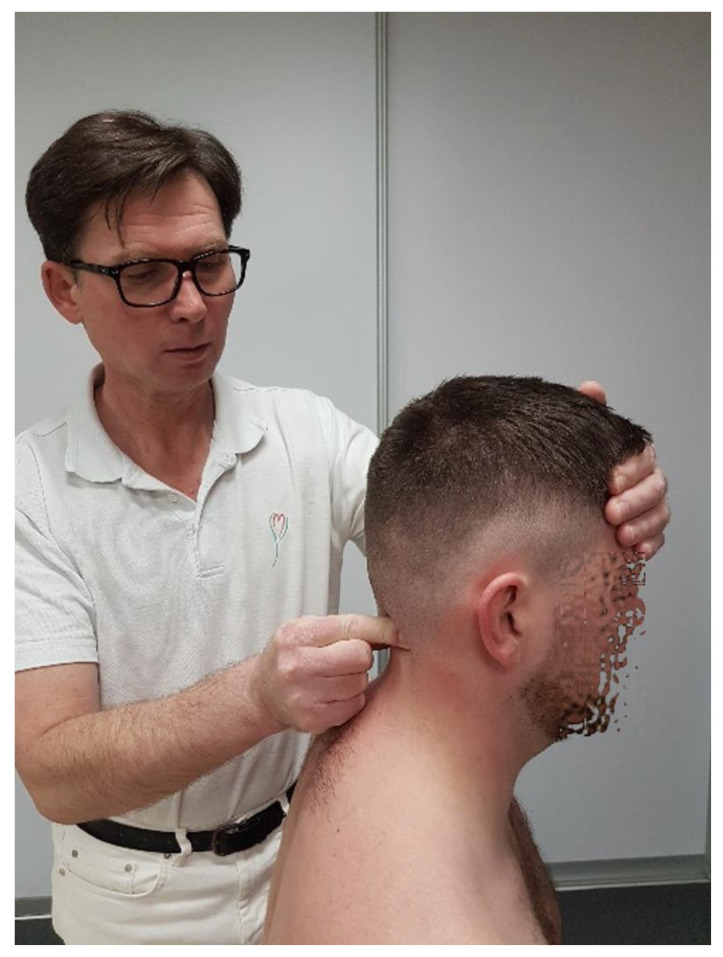
Provocative test for anterior instability at C1/C2 level (Sharp Purser Test).

In the second test, performed with the patient seated, the patient is asked to actively flex the head against resistance applied to the forehead while simultaneously controlling the position of the C2 spinous process. A characteristic sound and short subluxation of C2 indicate C1/C2 instability with transverse ligament insufficiency [22] (Figure 5).

Approximately 20% of the lateral flexion range of the cervical spine occurs in a coupled manner in the C0–C3 segments, making it difficult to isolate; therefore, unilateral increased lateral flexion in this area, especially with a positive lateral shear test, strongly correlates with alar ligament damage [23].

An additional provocative test for confirming alar ligament damage is the Mintken test (alar ligament insufficiency test). This test is performed by passively inducing lateral flexion and ipsilateral rotation at the C0/C1 level while stabilizing the C2 transverse process to prevent any movement of the C2 vertebra. In normal conditions, with adequate alar ligament function, the stabilization of the C2 vertebra restricts excessive motion at the C0/C1 level, resulting in a rapid increase in end-feel resistance during lateral flexion and rotation. A positive Mintken test occurs when there is an abnormal, unilateral increase in lateral flexion and rotation despite C2 stabilization, suggesting alar ligament damage. The alar ligament acts as the primary restraint for both rotation and lateral flexion at the C0/C1 level, and its insufficiency allows for excessive movement. The value of the individual provocative tests, including the Mintken test, remains a subject of debate, with reported specificity and sensitivity values ranging from 0.71 to 1.00 and from 0.33 to 0.96, respectively. As such, these tests are best utilized as initial screening tools in a grouped test approach to achieve the highest predictive value for diagnosing instability [24].

## 4. Radiological Criteria for Instability

In addition to manual tests, a distinguishing feature of true CCJ instability is the radiological image. In X-ray examinations in AP, open-mouth AP, and lateral projections, despite there being well-described reference points and lines, it is not always possible to effectively determine the degree of instability, especially in cases of neck shortening, head settling, and postural defects. Much clearer images are obtained using computed tomography (CT), where bony reference points for measurements can be very precisely identified on individual sections. The preferred method for identifying traumatic soft tissue injuries, especially alar and transverse ligament complex injuries, is magnetic resonance imaging (MRI) [25].

**Figure 6:**
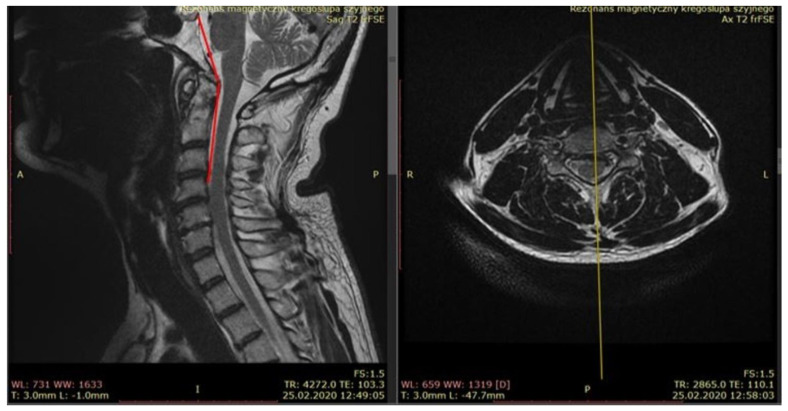
CXA in magnetic resonance imaging (MRI).

MRI can also be used to verify manual tests used to assess individual ligaments, quantitatively showing the range of segment displacement during anterior shear or axial traction tests (for atlantooccipital membrane stability) [21]. Regardless of the imaging method, the following criteria for CCJ instability have been adopted:

- A change in the distance from the Basion to the posterior wall of C2 > 6 mm (Basion Atlas Interval, BAI)—an increased distance indicates anterior subluxation of C0/C1.
- A change in the distance from the Basion to the apex of the dens > 5 mm (Basion Dens Interval, BDI)—an increased distance indicates distraction.
- A change in the distance between the atlas and the dens > 2 mm (Atlas Dens Index, ADI)—a change of up to 4 mm in children is normal; an increased distance indicates transverse ligament insufficiency.
- A change in the distance from the posterior aspect of the dens to the posterior aspect of the C1 arch < 13 mm—a decreased distance indicates spinal canal narrowing.
- A change in the distance between the lateral masses of C1 > 7 mm—AP open-mouth projection on X-ray; an increased distance indicates rupture of the C1 arch.
- Axial rotation C1/C2 > 45 degrees to each side—transverse projection on CT—an increased range of rotation indicates instability.
- Axial rotation C0/C1 > 8 degrees to each side—transverse projection on CT—an increased range of rotation indicates instability.
- Any crossing of the McRae line by the den’s apex, crossing of the McGregor line by the den’s apex > 4 mm, or crossing of the Chamberlain line > 6 mm indicates basilar invagination.
- The ratio of the distance from the Basion (B) to the posterior arch of the atlas (C) to the distance between the anterior aspect of the C2 dens (A) and the Opisthion (O), known as the Powers ratio, where BC/AO > 1 (normal < 1) indicates C1/C2 instability.
- Atlanto-occipital index (AOI) > 1 mm—an increased distance suggests distraction.
- Clivo-axial angle (CXA), which is the angle between the bony part of the skull base and the posterior part of the C2 dens; a normal range is very wide in the range of 139°–172°, depending on the head position (neutral position is in the range of 150–165°), but an angle below 135° may cause compression of the anterior brainstem and medulla oblongata (Figure 6).
- Space available for the cord (SAC) at C1 level > 14 mm.

It should be noted that a normal cervical X-ray does not exclude the presence of AOJ and AAJ injuries, and in the study by Griffen et al. [26], CT identified injuries requiring treatment in 32% of cases. Conversely, a pathological change visible in the joint (e.g., degenerative process) or even evident segment subluxation does not necessarily confirm the presence of a pain generator at that location. Among patients with RA with C1/C2 subluxation awaiting surgery, up to 50% are asymptomatic, and the incidence of occipital, frontal, retro-orbital pain, and radicular pain in the limbs is similar in patients with or without cervical subluxation (54% vs. 43%; 17% vs. 31%; 25% vs. 24%; and 47% vs. 48%, respectively) [27].

## 5. Symptomatology of CCJ Instability

One of the most common clinical features of CCJ instability is cervical or headache pain resulting from irritation of the articular branches of the spinal nerve and referred pain. For the AOJ and AAJ, referred pain includes the suboccipital, retroauricular, occipital, parietal, retro-orbital, frontal, and mandibular areas. It is assumed that cervical pain originates from the joints in 27–63% of cases, with the C2/C3 and C5/C6 joints being considered the most common sources [28].

The topography of this pain has been well described thanks to diagnostic and provocative injections, as there is no pathognomonic symptom in history or clinical examination that definitively indicates AOJ or AAJ damage as the definitive source of chronic neck and head pain (also through interaction with the spinal nucleus of the trigeminal nerve) [29].

The C2/C3 joint accounts for cervicogenic headaches in 27% (95% CI 18–36%) of patients with cervical pain and 53% (95% CI 37–68%) among those who suffer from headache as the dominant symptom [30].

However, headache or neck pain is not everything. The symptomatology of CCJ instability encompasses a very wide range of symptoms resulting from conflict with central nervous system structures, cerebrospinal fluid circulation disorders, compression and irritation of vertebrobasilar circulation vessel walls, and restrictions in venous blood outflow from the skull.

The complexity of symptomatology is evidenced by the set of symptoms associated with anterior brainstem and medulla oblongata compression in patients with congenital ligamentous laxity classified for surgical treatment, as presented by Henderson et al. [31]. These symptoms include headaches (100%), fatigue (100%), dizziness (dizziness) (100%), muscle pain (95%), upper limb weakness (90%), joint pain (85%), neck pain (85%), balance disorders (balance) (85%), night waking (85%), memory disorders (80%), gait disorders (80%), upper limb paresthesias (75%), cold hands and feet (75%), lower limb paresthesias (75%), learning disorders (65%), speech disorders (60%), swallowing and choking disorders (55%), daytime urinary frequency (more often than every 2 h) (60%), nighttime urinary frequency (more often than two times per night) (55%), Gastro Esophageal Reflux Disease (GERD) (55%), Irritable Bowel Syndrome (IBS) (50%), muscle tremors (40%), fainting (35%), back sensory disorders (30%), sleep apnea (25%), visual disturbances (75%), lower limb weakness (65%), and rotatory dizziness (vertigo) (65%).

Another very unpleasant symptom for the patient is the feeling of a bobbing head, the so-called “bobble head” symptom—a subjective feeling of a lack of control over the head position, causing disproportionate muscle effort and overstimulation of mechanoreceptors (true “bobble head doll syndrome” is associated with a third ventricle cyst and hydrocephalus in children). Patients also report head clicking, rubbing, crepitus often associated with swallowing movements, chronic muscle fatigue, contractures, and dystonias requiring constant position changes, as a static position causes head settling and worsening symptoms.

The sensation of an unstable head is accompanied by the symptom of a “pumped head”—an unpleasant sensation of increased intracranial pressure due to cerebrospinal fluid circulation disturbance and venous blood outflow impairment from the skull via the jugular foramen, most often resulting from skull settling (narrowing of the atlanto-occipital space with fixed extension of the C0/C1 joints). These symptoms are exacerbated by the Valsalva maneuver, as well as by laughter, crying, sneezing, coughing, defecation, and any effort. This situation leads to medulla oblongata and spinal cord compression; cerebellar tonsil compression, which can manifest as headaches; decreased intellectual abilities; concentration disorders; memory and association disorders (foggy brain); chronic fatigue; and visual disturbances (blurred vision and afterimages).

Compression of the vertebral arteries at the two anatomical bends (the V3 and V4 segments between C1/C2 and C0/C1), most often resulting from skull settling with an extension component (extension dysfunction hinders the posterior glide of the occipital condyles needed to initiate flexion movement at the C0/C1 level), causes vertebrobasilar insufficiency (VBI). VBI symptoms, known mnemonically as the 5 Ds (dizziness, diplopia, dysarthria, dysphagia, and drop attacks) and the 3 Ns (nausea, numbness, and nystagmus), constitute absolute contraindications to cervical spine manipulation.

Widely used VBI provocative tests involving extreme neck positions in the direction of compressing or stretching the vertebral arteries (cervical spine extension with rotation or cervical spine flexion with rotation) with the observation of the above-mentioned symptoms should be performed in patients with suspected CCJ only after ruling out obvious instability of this level; otherwise, they may yield false positive results.

The complex symptomatology of VBI may be evidenced by the effect of medulla oblongata, pons, and cerebellum ischemia due to the compression or embolization of the posterior inferior cerebellar artery (lateral medullary syndrome or Wallenberg syndrome). It includes VIII nerve damage—which presents as hearing loss; IX nerve damage—presenting as dysphonia and swallowing disorders; X nerve damage—presenting as dysphagia or hoarseness; XII nerve damage—presenting as dysarthria; spinothalamic tract damage—presenting as a loss of pain and temperature sensations on the same side of the face and the opposite half of the body; sympathetic tract damage—presenting as Horner’s syndrome on the same side, a low blood pressure, bradycardia, and reduced sweating; vestibular nuclei damage—presenting as dizziness, nystagmus, nausea, or vomiting; and cerebellar damage—presenting as dysarthria or ataxia.

The causes of VBI can include atherosclerotic changes, aneurysm, arteriovenous fistula, internal wall dissection, spasm, thromboembolic material, as well as mechanical causes—such as compression by CCJ instability, osteophyte of the C2 lateral mass, fascial band, tumor, blunt or penetrating trauma, and iatrogenic causes—and too violent traction and manipulation.

Another vascular conflict mechanism in CCJ instability is pressure on the internal carotid artery wall and additional irritation of cranial nerves IX, X, and XI. Consequences include gastrointestinal symptoms, such as constipation, diarrhea, peristalsis disorders, and abdominal pain (X nerve); systemic inflammatory response syndrome (SIRS), a type of excessive inflammatory response with the activation of pro-inflammatory cytokines, mast cell activation (Mastocyte Cell Activation Syndrome, MCAS), a type of anaphylactic reaction with edema, urticaria, blood pressure drop, difficulty breathing, diarrhea, and autoimmune reactions (including multi-joint edema, anxiety reactions), Eustachian tube dysfunction (presenting as tinnitus, vertigo, Meniere’s disease, dizziness, and swallowing disorders) (IX nerve); and cervical dystonia and torticollis (XI nerve) [32].

A reduction in the CXA can be attributed to cerebellar, brainstem, and spinal cord center disturbances due to cerebrospinal fluid flow disruption and constant excessive adherence of the ventral surface of the medulla oblongata to the bony wall. Clinical manifestations include cerebellar-type balance disorders—presenting as ground instability or pulling to one side, ataxia, or cranial nerve dysfunctions from V to XI; dysautonomia—presenting as orthostatic fainting and tachycardia, rhythm disturbances, heat intolerance, thirst (polydipsia), delayed gastric emptying (bloating), and cold hands and feet; and spinal cord center disturbances—including the spinal nucleus of the trigeminal nerve (facial pain and trigeminal neuralgia) and the spinal accessory nerve nucleus (cervical dystonia). All of these symptoms are also observed in Ehlers–Danlos and Marfan syndromes [33,34,35].

CCJ dysfunctions, however, cause many symptoms that cannot be classified as purely mechanical, and when both manual and radiological examinations lack clear evidence of mechanical failure of the ligamentous capsular system or even hypomobility with increasing muscle tension dominates, there must be an additional mechanism responsible. Evidence for the existence of this mechanism includes cases of patients after whiplash or iatrogenic cervical spine manipulations where symptoms typical of CCJ instability develop in the form of Whiplash-Associated Disorders (WADs) [36,37].

## 6. The Role of the Cervical Proprioception System

The key to understanding the concept of false (functional) CCJ instability may be the cervical proprioception system (CPS). This system plays a crucial role in controlling head and body positioning by supplementing the visual, vestibular, and cerebellar inputs. The CPS is composed of specialized sensory receptors, including mechanoreceptors located in the dura mater, fascia, joint capsules, and annulus fibrosus of the intervertebral discs (such as the Ruffini and Pacini corpuscles), as well as in tendons and muscle attachments (like Golgi tendon organs). These receptors provide feedback about the position and movement of the cervical spine, and their distribution is consistent across cervical segments. Additionally, muscle spindles, which are abundant in the suboccipital muscles and deep cervical muscles, play a crucial role in proprioception by detecting changes in muscle length and tension. Mechanoreceptors in the CPS are intricately connected to the connective tissue of the suboccipital muscles, the nuchal ligament, and the dura mater, allowing for continuous information exchange regarding tension. This connection, however, can also lead to the irritation of dura mater mechanoreceptors, which is thought to be one of the mechanisms underlying cervicogenic headaches [38].

In cases of whiplash or iatrogenic cervical spine manipulations, another mechanism may be activated. Increased mechanoreceptor activity is associated with chemical stimulation by low-grade inflammation (LGI), where local concentrations of interleukin 1, 6, and TNF-alpha not only lower the excitability threshold of peripheral pain receptors (peripheral sensitization) but also activate latent synapses on the thalamus to cingulate the gyrus pathway, typical of allodynia syndromes (central sensitization) [39,40,41].

CPS dysfunction is more pronounced in patients with cervical spondylosis and is proportional to the intensity of neck pain. Dysfunctions in this system may manifest as dizziness, postural instability, poor position sense, and balance disturbances, often mimicking vestibular disorders. In such cases, excessive and constant discharges from upper cervical segment mechanoreceptors, triggered by abnormal positioning due to tension in joint capsules, ligaments, or muscles, disrupt the integration of stimuli between the labyrinth, visual system, and cerebellum. This disharmony affects the vestibular nucleus, leading to inadequate vestibulospinal and vestibulo-ocular reactions, resulting in symptoms such as dizziness and nystagmus [42].

Quantitative testing of CPS dysfunction is performed through the joint position error (JPE) test. Numerous studies confirm the correlation between cervical pain intensity and JPE magnitude, and rehabilitation programs aimed at reducing the JPE have proven effective, opening new therapeutic possibilities for CPS-related disorders [43].

## 7. Differentiation of Dizziness and Postural Instability

The hyperactivity of craniocervical mechanoreceptors also leads to disturbances in the reflex regulation of postural muscle tone, manifesting as “general instability”. This type of dizziness should be differentiated from benign paroxysmal positional vertigo (BPPV), where dizziness is triggered by up or down head movements or turning in bed; usually, symptoms are not continuous and do not occur without movement, lasting no longer than several seconds, and after their resolution, hearing is not impaired (unlike in Meniere’s disease). Dizziness in BPPV, unlike in VBI, is not accompanied by fainting (drop attacks). The remaining features differentiating dizziness and postural instability in CPS disorders and other pathologies are presented in Table 1.

## 8. Management for CCJ Conditions

The management of CCJ conditions varies depending on the specific diagnosis—specifically, it depends on whether it involves ligamentous capsular hypermobility, functional postural disorders due to proprioceptive dysfunction, or true structural instability (Table 2).

For ligamentous capsular hypermobility without red flag symptoms, conservative treatment is the primary approach [44]. Patients are often treated with injections such as Platelet-Rich Plasma (PRP), Autologous Conditioned Serum (ACS), collagen, or prolotherapy to promote joint stability and reduce symptoms by diminishing LGI.

Additionally, proprioceptive training and biofeedback exercises help improve motor control and joint stability, while strengthening exercises targeting cervical muscles support long-term stability. Postural therapy and, in some cases, periodic use of orthoses may be recommended. Regular follow-up is crucial to monitor improvements in joint stability and adjust the treatment plan based on the patient’s progress [45].

For functional postural disorders caused by proprioceptive dysfunction, conservative treatments are also favored. This also includes injections such as PRP, ACS, or collagen, not only aiding tissue repair but also reducing LGI to desensitize proprioceptive output. Proprioceptive retraining exercises, often using biofeedback, help improve the patient’s postural control, while vestibular rehabilitation may be employed to address balance issues. Manual therapy and soft tissue techniques are used to relieve muscle tension and restore proper postural alignment [46].

Pharmacological treatments, such as antidepressants, myorelaxants, anticonvulsants, and anxiolytics, may also be prescribed to manage secondary symptoms [47]. Follow-up involves regularly reassessing proprioceptive function and postural control to ensure that rehabilitation is progressing as expected, and modifications to the therapy plan are made based on the patient’s response. In cases of true structural instability, especially when combined with red flag symptoms, surgical intervention (Occipitocervical Fixation) is typically required [48].

Patients are referred to a neurosurgeon or orthopedic specialist for stabilization procedures, such as fusion or decompression, depending on the severity of the misalignment or ligamentous damage as indicated by imaging tests. Before surgery, conservative management, including immobilization with a cervical collar, may be recommended to prevent further damage. After surgery, post-operative rehabilitation focuses on restoring strength, mobility, and proprioception through targeted exercises. Regular follow-ups are essential to ensure the long-term success of the surgical stabilization and to monitor for any potential complications.

## 9. Quality of Life in Patients with CCJ Instability

Chronic instability of the CCJ profoundly affects patients’ quality of life (QOL) [49]. Persistent pain, dizziness, and balance disorders contribute to significant physical limitations and functional impairments. Additionally, the psychological burden, including chronic stress, anxiety, and depression, often exacerbates the overall health condition [50]. The effective management of both true and functional CCJ instability is crucial for alleviating symptoms, reducing the psychological impact, and enhancing the QOL for these patients [51]. Multidisciplinary approaches that include physical therapy, psychological support, and medical interventions are essential in addressing the complex needs of patients suffering from chronic CCJ instability [29]. The intricate and multifaceted nature of CCJ instability, encompassing biomechanical, neurophysiological, and vascular aspects, underscores the necessity of comprehensive diagnostic and therapeutic strategies. Moreover, recognizing the substantial impact of CCJ instability on patients’ QOL highlights the importance of holistic treatment approaches. These approaches not only aim to stabilize anatomical structures but also address the functional, psychological, and social dimensions of the patient’s well-being. Integrating QOL considerations into clinical practice is paramount for improving patient outcomes and fostering a better understanding of the chronic nature of CCJ instability.

## 10. Summary

Distinguishing between true (structural) and functional instability has critical clinical implications that directly affect both diagnosis and treatment strategies. True instability, characterized by ligamentous laxity, bone deformities, or structural deficits, often requires surgical intervention to prevent further deterioration, neurological deficits, or life-threatening conditions such as brainstem or spinal cord compression. The identification of true instability necessitates a prompt and targeted approach, often involving radiological imaging, invasive testing, and surgical stabilization.

Recognizing the differences between structural and functional instability is critical in clinical decision making as the management strategies differ significantly. Structural instability often necessitates surgical intervention, whereas functional instability can be managed with conservative approaches.

On the other hand, functional instability involves hyperactivity of the proprioceptive system without clear structural deficits, often manifesting as increased muscle tone, dizziness, balance issues, and psychological distress. Patients with functional instability may not show positive findings on imaging or standard provocative tests, which makes clinical diagnosis more challenging. The management of functional instability typically focuses on conservative treatments, such as physical therapy, biofeedback, and proprioceptive retraining, to restore functional balance and reduce symptoms.

The ability to distinguish between these forms of instability allows for more personalized treatment plans. For example, patients with true instability may experience substantial relief from surgical interventions, whereas those with functional instability may benefit more from rehabilitation strategies. Additionally, failure to accurately differentiate these conditions can lead to ineffective treatments—such as unnecessary surgery for functional cases or insufficient rehabilitation for structural instability—resulting in prolonged patient suffering and delayed recovery.

Furthermore, the psychological impact on patients with functional instability can be profound, as these individuals often feel invalidated or misunderstood due to the absence of clear structural abnormalities in diagnostic tests. Effectively addressing this psychological burden requires a comprehensive, multidisciplinary approach that integrates mental health support with physical rehabilitation strategies. By recognizing the distinct nature of both true and functional instability, clinicians can enhance diagnostic precision and tailor treatment plans to ensure that patients receive the most suitable and effective care. This holistic approach not only promotes better symptom management but also contributes to improved long-term outcomes and a higher quality of life for patients.

Diagnosing functional instability presents several unique challenges due to the absence of obvious structural abnormalities on radiological imaging or standard mobility tests. Patients often present with symptoms such as dizziness, balance disturbances, and muscle hyperactivity, which can mimic other conditions and complicate the diagnostic process. Moreover, the subjective nature of many symptoms associated with functional instability, such as generalized instability or proprioceptive dysfunction, can lead to misdiagnosis or underestimation of the condition. To improve diagnostic accuracy, clinicians should adopt a multidisciplinary approach that includes a combination of detailed patient history, clinical examination, and functional tests specifically designed to assess proprioceptive and neuromuscular control. Incorporating advanced diagnostic techniques, such as joint position error testing, dynamic balance assessments, and biofeedback evaluations, can provide a more comprehensive picture of the patient’s functional limitations. Table 3 summarizes the diagnostic criteria for both structural and functional instability of the CCJ, highlighting differences in symptoms, diagnostic methods, and management approaches.

In conclusion, the distinction between true (structural) and functional instability of the CCJ is paramount for effective patient management. Recognizing and accurately diagnosing these two forms of instability ensures that patients receive the most appropriate interventions, whether they involve surgical correction for structural deficits or conservative management for functional impairments. Misdiagnosis or failure to differentiate between the two can lead to suboptimal treatment, prolonged patient suffering, and a diminished quality of life. As such, improving diagnostic accuracy for both types of instability is crucial for optimizing patient care and long-term outcomes. A comprehensive, multidisciplinary approach to diagnosis and management is essential to address the full spectrum of CCJ instability.

## Figures and Tables

**Table 1:** Differentiation of dizziness and postural instability.

Type of Dizziness	Character of Dizziness	Duration	Symptom Provocation	Symptom Reduction
Vestibular (Infectious Origin)	Vertigo, carousel-like movement, elevator ride, lateropulsion, vegetative symptoms, nausea and vomiting, clogged ear, hearing loss	Seconds to chronic	Independent of head position	Antibiotics, antiviral drugs, steroids, rehabilitation
Cerebellar, Posterior Columns, Polyneuropathy	Swaying, ground instability, feeling drunk, feeling of empty ground without visual control, no visual or auditory disturbances	Chronic	Upright position and exclusion of visual control	Visual control inclusion, lying position
CCJ Instability, CPS Dysfunction	General instability, swaying, ground instability, feeling drunk, feeling of empty ground, additional symptoms like dizziness, difficulty associating (dizziness), headaches, TMJ dysfunctions, tinnitus, dysautonomia, cranial nerve symptoms, visual disturbances	Seconds to chronic	Head position, flexion or extension (protrusion) in upper cervical segment, axial traction, whiplash, trunk rotation repetition (fixed head positioning while chair is mobile)	Orthopedic collar immobilization, “mechanical silence”, anti-inflammatory drugs or injections, muscle relaxants, anticonvulsants, antidepressants, positional training (biofeedback), manual therapy
Vascular—VBI	Swaying, ground instability, feeling drunk, feeling of empty ground, additional symptoms like visual disturbances, nystagmus, facial paresthesias, nausea, drop attacks	A few minutes with a period of slow resolution	Head position, combination of flexion/rotation, extension/rotation, axial traction, rapid body position change	Neutral neck position, drugs to improve cerebral circulation
Vascular—Vascular Diseases (Migraine), Orthostatic, Heart Rhythm Disorders, Stroke	Feeling of sliding and fainting, paresthesias, numbness of lips, visual disturbances of field loss type (ischemia), headache or eye pain, aura	Seconds—orthostatic; hours—migraine; chronic—stroke	Migraine triggers, dehydration, hormonal disorders	Antimigraine drugs, antiarrhythmics, hormones, hydration, rehabilitation
Phobia	Situational sudden vertigo or “leg cut-off”, vegetative symptoms	Seconds	Height, speed, movement amplitude changes	Assistance
BPPV	Head position-dependent, no visual or auditory disturbances	Seconds with a period of slow resolution	Head position	Hallpike maneuver

**Table 2:** Management algorithm for CCJ conditions.

Condition	Diagnosis	Management	Follow-Up
Ligamentous-Capsular Hypermobility	-Clinical history: non-specific neck pain, the feeling of a “heavy” head, periodic blocking, the habit of self-manipulation, hypermobility	-Conservative treatment: Platelet Rich Plasma (PRP), Autologous Conditioned Serum (ACS), collagen, prolotherapy injections	-Regular monitoring for improvement in joint stability and symptom management
	-Physical examination: mobility and tissue compliance tests, soft end-point	-Proprioceptive training, biofeedback	-Periodic evaluation to adjust therapy
	-Radiological imaging: no significant findings but may use hyperextension/flexion radiographs		
		-Strengthening exercises targeting cervical muscles and postural therapy in case of periodic orthosis	
Functional Postural Disorders (Proprioceptive Dysfunction)	-Clinical history: dizziness, imbalance issues, muscle hyperactivity, postural sway	-Conservative treatment: Platelet Rich Plasma (PRP), Autologous Conditioned Serum (ACS), collagen, prolotherapy injections	-Reassessment of proprioceptive function and postural control
	-Physical examination: proprioceptive tests, joint position error testing	-Proprioceptive retraining, biofeedback	-Monitor patient’s progress through periodic evaluation
	-Imaging: normal or minimal findings	-Vestibular rehabilitation (if applicable)	
		-Manual therapy: soft tissue release techniques for muscle tension and postural control therapy, joint mobilization Pharmacology: antidepressant, myorelaxants, anticonvulsant, anxiolytics	
True Structural Instability	-Clinical history: severe symptoms, neurological deficits	-Surgical referral: neurosurgery or orthopedic department	-Post-surgical rehabilitation: strengthening, mobility, and proprioception exercises
	-Physical examination: provocative tests (e.g., Sharp Purser, Anterior Shear)	-Surgical stabilization, fusion, or decompression based on imaging and clinical findings	-Regular post-operative follow-up to ensure stabilization and prevent further complications
	-Imaging (X-ray, MRI, CT): signs of vertebral misalignment, ligamentous injury	-Pre-operative conservative management: cervical collar or immobilization to prevent worsening	

**Table 3:** Diagnostic criteria for CCJ instability.

Criteria	Structural Instability	Functional Instability
Definition	Physical defects such as ligamentous laxity, bone deformities, or structural abnormalities	Proprioceptive dysfunction with no clear structural abnormalities on imaging
Primary Symptoms	Neurological deficits, abnormal movement patterns, chronic neck pain, fixed deformities	Dizziness, imbalance, postural sway, a feeling of disharmony of movements and disintegration of the body, muscle hypertension, tiredness
Diagnostic Criteria	Identified via radiological imaging (X-rays, CT, MRI), positive mobility tests, and provocative tests indicating ligament damage	Diagnosed through proprioceptive and neuromuscular control assessments, joint position error testing, and dynamic balance tests.
Key Diagnostic Tests	Sharp Purser Test, Anterior Shear Test	Joint position error testing, dynamic balance assessments
Provocative Tests	Positive for ligament damage or vertebral misalignment	Proprioceptive dysfunction detected through dynamic assessments
Management Approach	Surgical intervention: fusion, decompression, stabilization	Conservative therapy: injections, physical therapy, proprioceptive retraining, pharmacological support
Expected Outcome	Restored structural stability, prevention of further damage	Improved symptom management, enhanced proprioceptive function

## Data Availability

The data are contained within the article.
